# Lipid Transfer–Dependent Endosome Maturation Mediated by Protrudin and PDZD8 in Neurons

**DOI:** 10.3389/fcell.2020.615600

**Published:** 2020-12-15

**Authors:** Michiko Shirane

**Affiliations:** Department of Molecular Biology, Graduate School of Pharmaceutical Sciences, Nagoya City University, Nagoya, Japan

**Keywords:** organelle, endoplasmic reticulum, endosome, membrane contact site, neuron, PDZD8, protrudin, lipid transfer

## Abstract

Endosome maturation refers to the conversion of early endosomes (EEs) to late endosomes (LEs) for subsequent fusion with lysosomes. It is an incremental process that involves a combination of endosome fusion and fission and which occurs at contact sites between endosomes and the endoplasmic reticulum (ER), with knowledge of the underlying mechanisms having increased greatly in recent years. Protrudin is an ER-resident protein that was originally shown to regulate neurite formation by promoting endosome trafficking, whereas PDZD8 is a mammalian paralog of a subunit of the yeast ERMES (ER-mitochondrial encounter structure) complex that possesses lipid transfer activity. A complex of protrudin and PDZD8 was recently found to promote endosome maturation by mediating lipid transfer at ER-endosome membrane contact sites. This review focuses on the roles of the protrudin-PDZD8 complex in tethering of endosomes to the ER, in mediating lipid transfer at such contact sites, and in regulating endosome dynamics, especially in neuronal cells. It also addresses the physiological contribution of endosome maturation mediated by this complex to neuronal polarity and integrity.

## Introduction

Most intracellular organelles of eukaryotic cells communicate with the endoplasmic reticulum (ER) network through membrane contact sites (MCSs), at which the membranes of the ER and the interacting organelle come into close proximity and are tethered. MCSs are thus thought to function as intracellular synapses, where molecular information is exchanged.

Neurons are polarized cells that consist of two distinct portions, the somatodendritic compartment and the axon. Trafficking of endosomes along the axon toward its terminus plays an important role in axonal outgrowth directed toward target cells as well as in neurotransmitter release. Protrudin was first identified as a protein that promotes neurite outgrowth through regulation of directional endosome trafficking (Shirane and Nakayama, 2006; Shirane, 2019). The protrudin binding proteins VAP [vesicle-associated membrane protein (VAMP)–associated protein] and KIF5 (kinesin heavy chain 5) also contribute to endosome trafficking (Saita et al., 2009; Matsuzaki et al., 2011), and protrudin-dependent regulation of such trafficking is mediated at MCSs between the ER and late endosomes (LEs) (Raiborg et al., 2015).

Axonopathy, a type of neurodegeneration, is caused by damage to the axon of neurons. The longest axons in the central nervous system of mammals are located in the corticospinal tract, which is the neural circuit responsible for voluntary movement. Hereditary spastic paraplegia (HSP) is an axonopathy in which upper motor neurons in the corticospinal tract undergo degeneration (Blackstone, 2012; Hubner and Kurth, 2014). Many HSP-related proteins—including spastin, REEPs (receptor expression–enhancing proteins), reticulons, atlastins, as well as protrudin—have been identified (Mannan et al., 2006; Hashimoto et al., 2014; Pyle et al., 2015; Connell et al., 2020). Most such proteins contain a hairpin domain, which is a key determinant of membrane structure and function in the ER. HSP-related proteins have recently been implicated in the regulation of endosome maturation at ER-endosome MCSs (Allison et al., 2017), although the physiological role and regulatory mechanisms of such maturation remain to be fully elucidated. A new study has now revealed that protrudin-dependent lipid transfer from the ER to endosomes promotes endosome maturation at ER-endosome MCSs (Shirane et al., 2020b). Protrudin-deficient mice show no signs of axonopathy, however, but instead manifest an abnormal behavioral phenotype (Shirane et al., 2020a), suggesting that protrudin might play an important role in normal neuronal development and behavior.

In this review, I summarize what is known of the mechanism responsible for regulation of endosome maturation by protrudin and its relation to the pathogenesis of neurological disease. We also address the role of PDZD8 (PDZ domain–containing protein 8), a protrudin-interacting protein, in the lipid transfer process underlying endosome maturation. Finally, we discuss the contribution of the protrudin-PDZD8 complex and its lipid transfer function to the maintenance of neuronal polarity and integrity.

## Protrudin Regulates Directional Endosome Trafficking

Protrudin was originally discovered as a protein of unknown function that interacts with FK506 binding protein 38 (FKBP38) (Shirane and Nakayama, 2003; Shirane et al., 2008; Saita et al., 2013). Forced expression of protrudin in cultured cells resulted in pronounced membrane deformation followed by the formation of long protrusions, hence the designation “protrudin” (Shirane and Nakayama, 2006). Protrudin is an ER-resident protein that harbors various functional domains including a Rab11 binding domain (RBD11), two transmembrane (TM) domains, a hairpin (HP) domain, a low complexity region (LCR), a two phenylalanine in an acidic tract (FFAT) motif, a coiled-coil (CC) domain, and a Fab1, YOTB, Vac1, and EEA1 (FYVE) domain. These structural characteristics underlie the multiple functions of protrudin in the regulation of organelle dynamics including directional endosome trafficking and ER morphogenesis.

**FIGURE 1:**
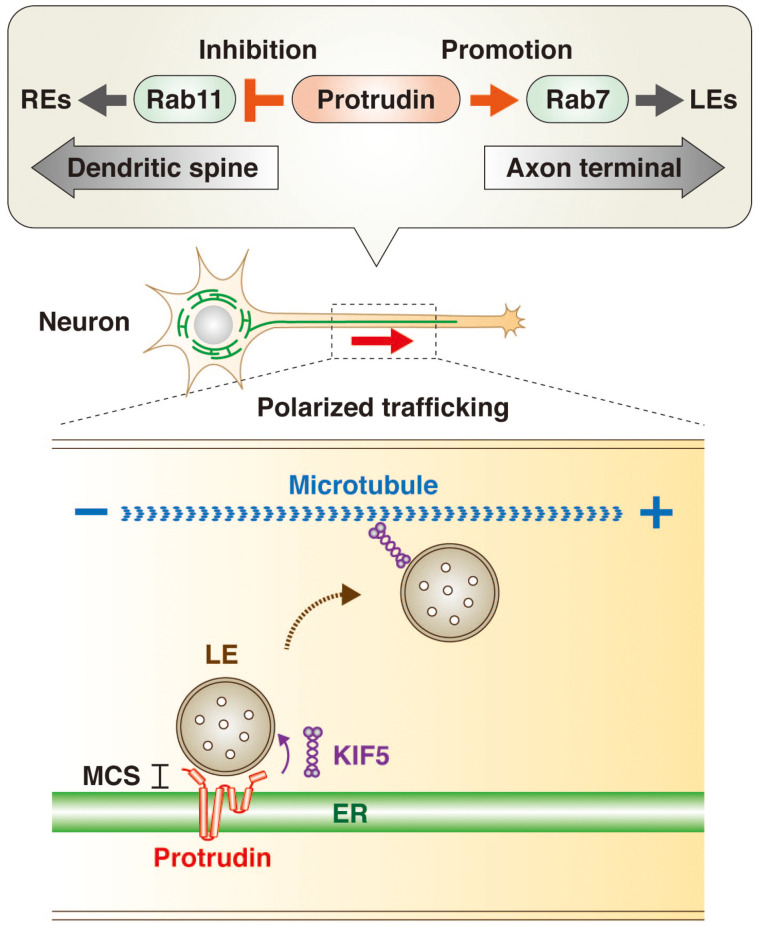
Protrudin regulates endosome dynamics underlying directional endosome trafficking in neuronal cells through interaction with Rab GTPases and KIF5. Protrudin interacts with the GDP-bound (inactive) form of Rab11 and thereby inhibits RE trafficking toward dendritic spines. It also interacts with the GTP-bound (active) form of Rab7 and thereby promotes LE trafficking toward the axon terminal (upper insert). In addition, protrudin tethers LEs to the ER at MCSs, where it loads LEs with the microtubule-dependent motor protein KIF5. The subsequent release of the KIF5-loaded LEs to microtubules results in their transport from the minus end to the plus end of the microtubules in the direction of the axon terminal (lower insert).

Rab GTPases are master regulators and markers of organelle identity in the endocytic pathway (Zerial and McBride, 2001; Stenmark, 2009; Wandinger-Ness and Zerial, 2014). The transformation of early endosomes (EEs) to LEs is accompanied by a switch in associated Rab protein from Rab5 to Rab7, whereas recycling endosomes (REs) are associated with Rab11. The GTP-bound (active) form of Rab11 promotes directional trafficking of REs from the apical to the basolateral domain of epithelial cells as well as from the axonal to the somatodendritic domain of neurons. In contrast, the GTP-bound form of Rab7 promotes LE trafficking toward the axon terminal in neuronal cells. Protrudin interacts with both the GDP-bound (inactive) form of Rab11 (Shirane and Nakayama, 2006) and the GTP-bound form of Rab7 (Raiborg et al., 2015) and appears to function as a hub for endosomal trafficking by inhibiting Rab11-dependent RE trafficking and promoting Rab7-dependent LE trafficking (Figure 1). Protrudin thus increases the supply of membrane to the tip of neurites by facilitating axonal transport of membrane-containing endosomes, resulting in polarized neurite outgrowth.

Protrudin also interacts with the microtubule-dependent motor protein KIF5, which mediates anterograde cargo trafficking along microtubules of axons in the plus-end direction (Saita et al., 2009; Matsuzaki et al., 2011; Ohnishi et al., 2014). Indeed, protrudin was recently shown to facilitate loading of the endosome membrane with KIF5 at ER-LE MCSs, with the KIF5-loaded endosomes then being released for interaction with microtubules (Raiborg et al., 2015; Figure 1).

VAP has also been identified as a protrudin binding protein (Saita et al., 2009), with the major sperm protein (MSP) domain of VAP mediating interaction with the FFAT motif of protrudin. VAP resides at MCSs and is implicated in lipid transfer processes (Helle et al., 2013; Phillips and Voeltz, 2016; Salvador-Gallego et al., 2017; Wu et al., 2018). Both the interaction of protrudin with VAP and the induction of process formation by protrudin were found to be attenuated by mutation of the FFAT motif of protrudin. Knockdown of VAP also resulted in mislocalization of protrudin and in inhibition of neurite outgrowth induced by nerve growth factor in PC12 pheochromocytoma cells, suggesting that binding to VAP is indispensable for the regulation of endosome trafficking by protrudin (Saita et al., 2009).

The precursor mRNA for protrudin is alternatively spliced, resulting in the generation of mature transcripts for two different isoforms of protrudin, designated L (long) and S (short) (Ohnishi et al., 2014). Protrudin-S appears to be ubiquitously expressed in mammalian tissues, whereas protrudin-L is expressed specifically in neuronal cells. Relative to protrudin-S, protrudin-L contains an additional seven amino acids encoded by exon L. These additional residues are located adjacent to the FFAT motif, which mediates binding to VAP, with the result that the binding affinity of protrudin-L for VAP is greater than that of protrudin-S. Protrudin-L is thus more effective at promoting neurite outgrowth than is protrudin-S. The neural-specific splicing regulator SRRM4 was found to promote the splicing of protrudin pre-mRNA to yield protrudin-L mRNA (Ohnishi et al., 2017).

## Endosome Maturation at ER-Endosome MCSs

Endosomes play an important role in fundamental cellular activities. A subset of EEs formed by endocytosis through invagination of the plasma membrane undergoes conversion to LEs. LEs contain multiple intraluminal vesicles (ILVs) that are derived from luminal invaginations of the LE membrane, and so they are also known as multivesicular bodies (MVBs) (Huotari and Helenius, 2011). Endosome maturation is the process by which EEs are converted to LEs for fusion with lysosomes, which degrade endocytosed material for reutilization. It is an incremental process, with the vesicles on this continuum being collectively referred to as endolysosomes (LyLEs) (Hong et al., 2017). In addition to this degradation pathway dependent on the endocytic machinery, another subset of EEs is delivered to a recycling pathway, in which the EEs are converted to REs for recycling of material back to the plasma membrane. Some LEs also undergo exocytosis, resulting in the release of their ILVs as extracellular vesicles known as exosomes, which play a key role in intercellular communication (Colombo et al., 2014).

The membrane dynamics of endosome maturation are largely attributable to a combination of endosome fission and fusion (Rowland et al., 2014; Allison et al., 2017; Hoyer et al., 2018). Such fission and fusion as well as the transport of LEs are thought to depend on lipid transfer at ER-endosome MCSs (Johansson et al., 2005, 2007). However, the factors that tether endosomes to the ER at MCSs and the mechanism underlying such lipid transfer from the ER to endosomes had been mostly unknown until recently (Kobuna et al., 2010; Huotari and Helenius, 2011; van der Kant and Neefjes, 2014; Dong et al., 2016; Wong et al., 2018).

## Relation of ER-Endosome MCSs to the Mechanism of Axonopathy

**FIGURE 2:**
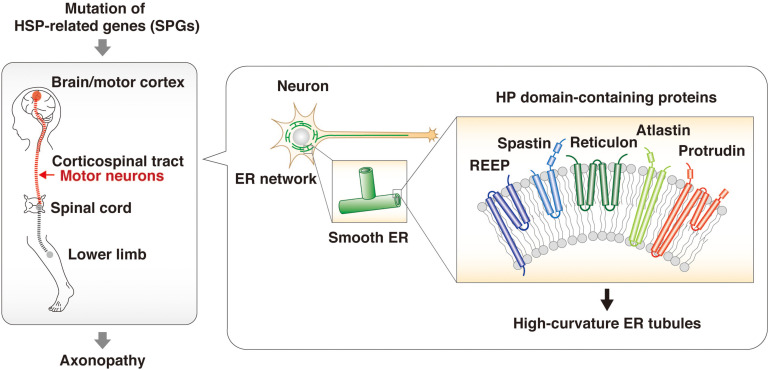
HSP-related proteins regulate ER morphology through HP domains. HSP is an axonopathy that is caused by mutation of various genes and which is characterized by the degeneration of motor neurons in the corticospinal tract and consequent progressive spasticity and weakness of the lower limbs. The proteins encoded by such HSP-related genes—including spastin, REEPs, atlastins, and reticulons as well as protrudin—contain an HP domain. This hydrophobic wedge-shaped domain inserts into the cytosolic side of the ER membrane, resulting in bending of the membrane bilayer and the formation of high-curvature tubules. The HSP-associated mutant forms of these proteins give rise to structural defects in the smooth ER and an abnormal ER network.

Mutations of the protrudin gene (*ZFYVE*27) are responsible for a subset of cases of HSP (Mannan et al., 2006; Zhang et al., 2012; Hashimoto et al., 2014; Hubner and Kurth, 2014; Powers et al., 2017; Fowler et al., 2019). The genes mutated in different subsets of individuals with HSP are referred to as spastic paraplegia genes (SPGs), and protrudin is therefore also referred to as SPG33. The predominant clinical features of HSP are progressive spasticity and weakness of the lower limbs caused by degeneration of the long axons of motor neurons in the corticospinal tract. Proteomics analysis of the brain of neuron-specific protrudin transgenic mice showed that protrudin associates with multiple HSP-related proteins including myelin proteolipid protein 1 (SPG2), atlastin 1 (SPG3A), REEP1 (SPG31), REEP5, KIF5A (SPG10), KIF5B, KIF5C, and reticulons 1, 3, and 4 (which are similar to reticulon 2, or SPG12) (Hashimoto et al., 2014). Protrudin was also found to bind to spastin (SPG4) (Mannan et al., 2006). These various HSP-related proteins contain an HP domain, a hydrophobic wedge-shaped structure whose insertion into the cytosolic side of the ER membrane results in bending of the membrane bilayer and the formation of high-curvature tubules (Shibata et al., 2006; Voeltz et al., 2006; Hu et al., 2008, 2009). The axonopathy associated with HSP has therefore been suggested to result from an abnormal ER morphology that affects the smooth ER network and increases susceptibility to ER stress (Hashimoto et al., 2014; Figure 2). HSP caused by mutation of the protrudin gene may thus be attributable to a dominant negative effect resulting from accumulation of the mutant protein in the ER membrane and consequent ER stress.

Neurons with HSP-associated mutations of the genes for spastin or REEP1 were recently found to manifest abnormal enlargement of LEs and lysosomal dysfunction as a result of defects in ER-endosome MCSs and impaired endosomal homeostasis (Allison et al., 2017; Lee et al., 2020). As described in more detail below, disruption of the protrudin-PDZD8 complex has also been shown to result in the formation of abnormal LEs as well as in disturbance of neuronal polarity and axonal degeneration (Shirane et al., 2020b). These findings implicate the protrudin-PDZD8 complex in regulation of endosome maturation at ER-endosome MCSs.

**FIGURE 3:**
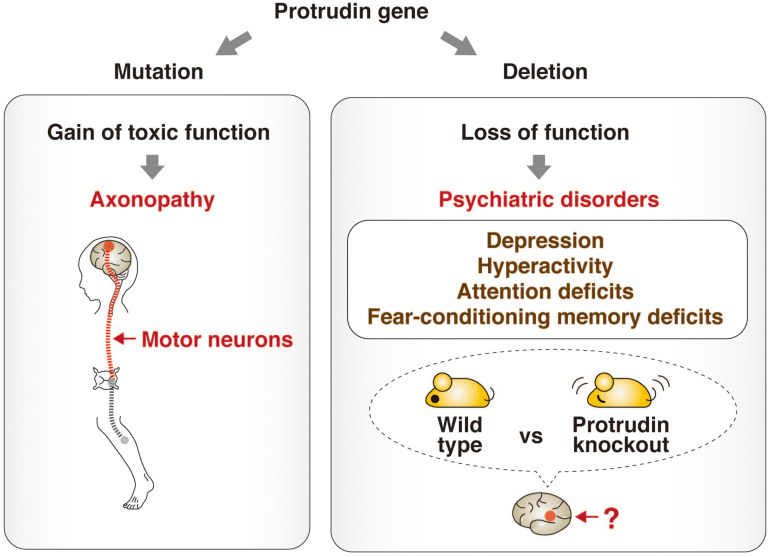
Phenotypes associated with point mutation or deletion of the protrudin gene. Mutations of the protrudin gene (*ZFYVE27*) in humans give rise to axonopathy as a result of degeneration of motor neurons caused by a gain of toxic function (left). In contrast, deletion of the protrudin gene in mice gives rise to psychiatric-like disorders including depression-like behavior with abnormalities in activity, attention, and cued fear-conditioning as a result of a loss of function (right). The pathophysiologic mechanism, including the identity of the affected brain regions and neuronal circuits, underlying such behavioral defects in protrudin-deficient mice remains to be determined, but these deficits suggest that protrudin might play an indispensable role in the nervous system.

A recent study has shed light on the physiological role of protrudin by subjecting protrudin-deficient mice to a comprehensive battery of behavioral tests (Shirane et al., 2020a). The protrudin-deficient mice showed no signs reminiscent of HSP, but instead manifested depression-like behavior with abnormalities in activity, attention, and cued fear-conditioning. Mutations of the protrudin gene therefore likely give rise to axonopathy as a result of a gain of toxic function, whereas protrudin nullizygosity gives rise to psychiatric-like disorders as a result of a loss of function. These findings suggest that protrudin might play an indispensable role in normal neuronal development and behavior (Figure 3).

## The Protrudin-PDZD8 Complex at ER-Endosome MCSs

**FIGURE 4:**
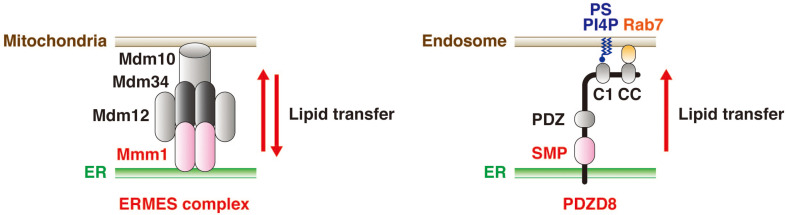
PDZD8 mediates lipid transfer from the ER to endosomes. The yeast ERMES complex—which contains Mmm1, Mdm12, Mdm10, and Mdm34—mediates the transfer of phospholipids between the ER and mitochondria in both directions (left). On the other hand, PDZD8, a mammalian paralog of Mmm1, mediates the transfer of several types of lipid from the ER to endosomes in a manner dependent on its SMP domain (right). The C1 domain of PDZD8 contributes to tethering of endosomes to the ER through its interaction with PS or PI4P in the endosome membrane. The CC domain of PDZD8 also supports such tethering through its interaction with the GTP-bound form of Rab7.

A differential proteomics analysis of brain extracts from wild-type and protrudin-deficient mice was performed to identify proteins that might function cooperatively with protrudin at ER-endosome MCSs. This analysis uncovered PDZD8, in addition to VAP-A and VAP-B, as a key binding partner of protrudin (Elbaz-Alon et al., 2020; Shirane et al., 2020b). An independent study also identified protrudin as a binding partner of PDZD8 (Elbaz-Alon et al., 2020). PDZD8 is a mammalian paralog of yeast Mmm1, a subunit of the ER-mitochondrial encounter structure (ERMES) complex. This complex mediates interaction between the ER and mitochondria and contributes to the biosynthesis of phospholipids by mediating lipid transfer in a manner dependent on the synaptotagmin-like mitochondrial lipid–binding protein (SMP) domain of Mmm1 (Figure 4; Kornmann et al., 2009). Although PDZD8 was also known to tether the ER and mitochondria and to regulate Ca^2+^ dynamics in neurons (Hirabayashi et al., 2017), it was only shown to possess lipid transfer activity after its identification as a binding partner of protrudin (Shirane et al., 2020b). Protrudin and PDZD8 form a stable complex at the ER membrane, with the abundance of protrudin being greatly diminished in the brain of PDZD8-deficient mice. Knockdown of PDZD8 in PC12 cells also resulted in a loss of protrudin that was dependent on the proteasome (Shirane et al., 2020b).

Immunofluorescence analysis by super-resolution microscopy revealed that, like protrudin, PDZD8 is located at MCSs between the ER and endosomes (Shirane et al., 2020b). PDZD8 colocalized to a markedly greater extent with the ER than with endosomes at these MCSs, consistent with the notion that it is an ER-resident protein that makes contact with endosomes. The number of ER-endosome contacts was also found to be reduced in cells depleted of protrudin or PDZD8 by RNA interference. Although such depletion of protrudin or PDZD8 also attenuated MCS formation between the ER and mitochondria, this effect was less pronounced than that on ER-endosome contacts. Furthermore, protrudin and PDZD8 showed a synergistic effect on formation of ER-endosome contacts (Shirane et al., 2020b).

PDZD8 also contains a CC domain that interacts with the GTP-bound form of Rab7, which localizes to LEs (Chavrier et al., 1990; Raiborg et al., 2015; Guillen-Samander et al., 2019). In addition, a recent study suggested that the protrudin-PDZD8 complex resides at a microdomain at which three organelles—the ER, endosomes, and mitochondria—come into contact with each other (Elbaz-Alon et al., 2020). However, further studies are needed to reveal the physiological function of such ER-endosome-mitochondrion contacts mediated by the protrudin-PDZD8 complex.

## PDZD8 Possesses Lipid Transfer Activity

MCSs mediate lipid transfer, Ca^2+^ homeostasis, and organelle dynamics. PDZD8 regulates Ca^2+^ dynamics in neurons, and it contains a SMP domain characteristic of the TULIP (tubular lipid-binding protein) superfamily of proteins that possess lipid transfer activity (Kopec et al., 2010; Watanabe et al., 2015; Alva and Lupas, 2016). Furthermore, as mentioned above, the yeast PDZD8 paralog Mmm1 mediates lipid transfer by the ERMES complex (Kornmann et al., 2009). A liposome–FRET (fluorescence resonance energy transfer) assay was therefore applied to determine whether PDZD8 also possesses lipid transfer activity. For this assay, donor liposomes were prepared by mixing rhodamine-labeled lipid and nitrobenzoxadiazole (NBD)–labeled lipid, with the result that NBD fluorescence was quenched by FRET. Extraction of lipids from the donor liposomes and their dispersal by PDZD8 would abolish such quenching and thereby allow the detection of NBD fluorescence. This assay revealed that phospholipids—including phosphatidic acid, phosphatidylserine (PS), phosphatidylethanolamine, and phosphatidylcholine—as well as ceramide and cholesterol were extracted from the donor liposomes by PDZD8 (Shirane et al., 2020b).

Lipid transfer between membranes comprises two steps, lipid extraction from the donor membrane and lipid insertion into the acceptor membrane. These steps are distinguishable by performance of the liposome-FRET assay in the absence or presence of acceptor liposomes. Such analysis showed that PDZD8 possesses only lipid extraction activity, with this activity presumably being unidirectional from the ER to other organelles *in vivo*. In addition, both the SMP and PDZ domains of PDZD8 were found to contribute to this lipid extraction activity. As a result of the insolubility of full-length PDZD8, the liposome-FRET assay was performed with a recombinant protein consisting of glutathione S-transferase (GST) fused to a form of PDZD8 lacking the TM domain [PDZD8(ΔTM)]. The mutant protein was thus unable to dock in the liposome membrane. This drawback was subsequently addressed by the addition of DGS-NTA(Ni), a conjugated phospholipid that binds the hexahistidine epitope tag, to the donor liposomes. Performance of the assay with His_6_-PDZD8(ΔTM) thus allowed association of the tagged PDZD8 protein with the liposome membrane. Under these conditions, PDZD8 also showed lipid extraction activity (Shirane et al., 2020b).

Although the typical C1 domain binds to diacylglycerol, PDZD8 possesses a C1 domain that was found to preferentially interact with PS and phosphatidylinositol 4-phosphate (PI4P) but not with diacylglycerol (Shirane et al., 2020b). By analogy to extended synaptotagmin (E-Syt) proteins (Giordano et al., 2013; Schauder et al., 2014; Saheki et al., 2016; Bian et al., 2018), the C1 domain of PDZD8 might tether the ER and endosomes by interaction with PS and PI4P enriched in the endosome membrane. Such ER-endosome tethering might be regulated by an intracellular signal that induces a conformational change of PDZD8, resulting in an increase in lipid extraction activity mediated by its SMP domain. The tethering is also promoted by interaction between the CC domain of PDZD8 and the GTP-bound form of Rab7 (Raiborg et al., 2015; Guillen-Samander et al., 2019). PDZD8 likely promotes lipid transfer *in vivo* (Figure 4), given that depletion of PDZD8 results in a decrease in the abundance of PS in endosomes of neurons (Shirane et al., 2020b). However, further experiments will be required to demonstrate definitively the physiological lipid transfer activity of PDZD8.

## Lipid Transfer Proteins at ER-Endosome MCSs

**FIGURE 5:**
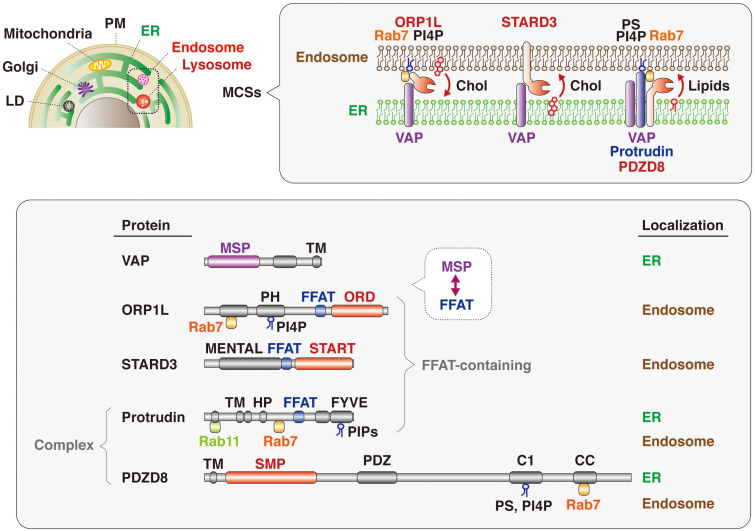
Lipid transfer proteins at ER-endosome MCSs. Many intracellular compartments—including endosomes, lysosomes, the plasma membrane (PM), mitochondria, the Golgi apparatus, and lipid droplets (LD)—are tethered to the ER at MCSs (upper). Representative protein complexes that tether the ER and endosomes are shown (upper insert). ORP1L, which is recruited to endosomes through PI4P and Rab7, interacts with the ER-resident protein VAP and transfers cholesterol (Chol) from endosomes to the ER. The endosome-resident protein STARD3 binds to VAP and transports cholesterol from the ER to endosomes. The protrudin-PDZD8-VAP complex, which is localized at the ER and tethers endosomes through interaction with Rab7 and phosphoinositides (PIPs), transfers lipids from the ER to endosomes. The domain structure and subcellular localization of these various proteins are also shown (lower). The MSP domain of VAP interacts with the FFAT motifs of lipid transfer proteins such as ORP1L and STARD3. Both ORP1 and STARD3 possess lipid transfer domains: ORD and START, respectively. Protrudin also has an FFAT motif, and PDZD8 harbors a lipid transfer–related SMP domain. Both protrudin and PDZD8 interact with Rab7 and PIPs associated with the endosome membrane.

The mechanism underlying lipid transfer at MCSs has been extensively studied, with multiple tethering factors and lipid transfer proteins having been identified, including those that function at MCSs between the ER and endosomes (Figure 5). VAP (VAP-A and VAP-B) is an ER-resident protein that tethers other organelles to the ER and plays a key role in lipid transfer. VAP interacts via its MSP domain with multiple proteins that contain an FFAT motif (Kaiser et al., 2005; Loewen and Levine, 2005; Saita et al., 2009; Huttlin et al., 2015). Mutations in the MSP domain of VAP-B (also known as ALS8) are responsible for a dominant form of amyotrophic lateral sclerosis (ALS) (Nishimura et al., 2004). FFAT motif–containing proteins that contribute to tethering and lipid transfer at MCSs include oxysterol binding protein (OSBP)–related proteins (ORPs), steroidogenic acute regulatory protein (StAR)–related lipid transfer (START) domain–containing proteins, phosphatidylinositol transporter protein (PITP) domain–containing proteins, and Sec14-like proteins. With regard to lipid transfer at ER-endosome MCSs, OSBP and ORPs transport lipids such as sterol and phosphoinositides at MCSs (Olkkonen and Li, 2013); OSBP together with SNX2 and VAP regulates endosome budding through control of actin nucleation and retromer function at ER-endosome MCSs (Dong et al., 2016); ORP1L transports cholesterol from endosomes to the ER in cooperation with VAP and Rab7 (Zhao and Ridgway, 2017; Ma et al., 2018); and ORP5 together with NPC1 mediates the exit of cholesterol from LyLEs at MCSs (Du et al., 2011). The START domain–containing protein STARD3 is anchored to the membrane of LEs and mediates cholesterol transport from the ER to endosomes in concert with VAP (Alpy et al., 2013; Wilhelm et al., 2017). VPS13C, which is associated with early-onset Parkinson’s disease, also tethers the ER and endosomes and transfers lipids at MCSs in cooperation with VAP and Rab7 (Lesage et al., 2016; Kumar et al., 2018; Gillingham et al., 2019).

It has also now been revealed that PDZD8, which harbors a lipid transfer–related SMP domain, interacts with the FFAT motif–containing protein protrudin as well as with Rab7. The protrudin-PDZD8 complex tethers the LyLE membrane to the ER and promotes lipid transfer from the ER to endosomes (Figure 5). The FYVE domain of protrudin is atypical in that the amino acid sequences responsible for binding to PI3P are not conserved and that it binds to several lipids such as PI(4,5)P_2_, PI(3,4)P_2_, and PI(3,4,5)P_3_ (Gil et al., 2012). Furthermore, Rab7 interacts with both protrudin and PDZD8 (Raiborg et al., 2015; Guillen-Samander et al., 2019). However, it remains an open question and warrants further investigation whether both protrudin and PDZD8 are bound to the endosomal membrane simultaneously. Protrudin, PDZD8, and Rab7 are all related to neurological disorders, with mutations in the protrudin gene giving rise to the axonopathy HSP (Mannan et al., 2006; Hashimoto et al., 2014), protrudin-deficient mice manifesting psychiatric-like disorders (Shirane et al., 2020a), mutations in the PDZD8 gene being a risk factor for posttraumatic stress disorder (PTSD) (Bharadwaj et al., 2018), and mutations in the Rab7 gene causing another axonopathy, Charcot-Marie-Tooth disease (CMT) (Verhoeven et al., 2003; McCray et al., 2010).

## Endosome Maturation Influences Neuronal Polarity and Integrity

The amounts of endogenous protrudin and PDZD8 are higher in the brain than in other tissues, suggesting that the protrudin-PDZD8 complex may function selectively in the nervous system. Depletion of protrudin or PDZD8 with the use of small interfering RNAs (siRNAs) in mouse primary neurons induced abnormal enlargement of LEs, with the resulting vesicles thus being designated abnormal large vacuoles (ALVs) (Shirane et al., 2020b). This phenotype caused by PDZD8 depletion was rescued by additional expression of an siRNA-resistant form of PDZD8 but not by that of a lipid extraction–deficient mutant [PDZD8(ΔSMP)]. The ALVs were also observed in neurons expressing PDZD8(ΔSMP) in the presence of endogenous PDZD8, likely as a result of a dominant negative effect of the mutant protein on the normal fission of LyLEs. The ALVs showed an aberrant multilamellar ultrastructure without ILVs. This phenotype was also highly reminiscent of that of neurons of spastin or REEP1 mutant mice (Allison et al., 2017).

Neurons depleted of PDZD8 showed a reduced axon length and increased somatodendritic area (Shirane et al., 2020b), reflecting impairment of cell polarity, and these abnormalities were similar to those of neurons derived from protrudin-deficient mice (Ohnishi et al., 2014; Shirane et al., 2020b). These observations thus suggested that the protrudin-PDZD8 complex is essential for the establishment of cell polarity in neurons.

**FIGURE 6:**
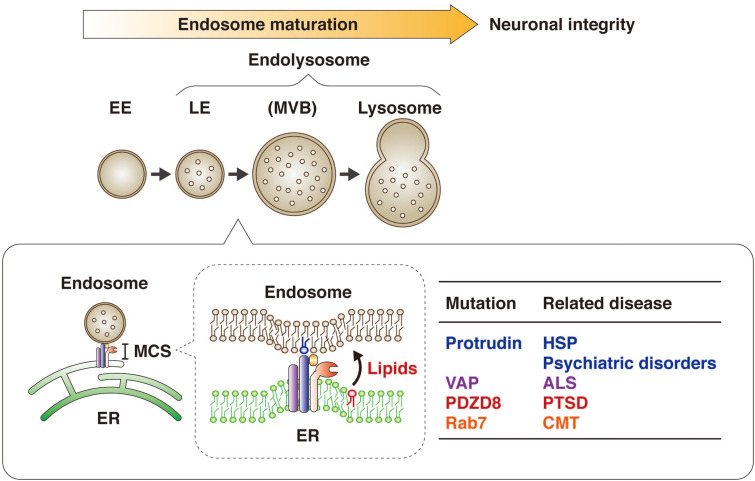
Protrudin regulates endosome maturation at ER-endosome MCSs in neurons and thereby maintains neuronal integrity. Protrudin interacts with VAP, PDZD8, and Rab7 at ER-endosome MCSs, at which lipids are transferred from the ER to endosomes in a manner dependent on the SMP domain of PDZD8, resulting in endosome maturation. Mutations of protrudin, VAP, PDZD8, or Rab7 gene are related to neurological disorders.

Defects in ER-endosome contacts induced by HSP-associated mutations of spastin or REEP1 result in lysosomal abnormalities in neurons (Allison et al., 2017). The fact that mutations of the protrudin gene also cause HSP suggested that the protrudin-PDZD8 system might contribute to maintenance of neuronal integrity. Neurons depleted of protrudin or PDZD8 indeed manifested a morphology consistent with axonal degeneration (Shirane et al., 2020b), including axonal thinning as well as dissociation of Tau1 from microtubules (Morris et al., 2011). This phenotype thus suggested that the protrudin-PDZD8 system protects neurons from axonal degeneration and is essential for neuronal integrity (Figure 6). Given that mutations of the PDZD8 gene have been associated with PTSD (Bharadwaj et al., 2018), further study of the physiological role of PDZD8 by analysis of PDZD8-deficient mice is warranted.

## Conclusion

I have here focused on the role of protrudin at ER-endosome MCSs in endosome maturation in neurons. Protrudin regulates endosome dynamics as well as ER structure, especially in neuronal cells. Mutations of the protrudin gene in humans give rise to the axonopathy HSP as a result of a gain of toxic function. Ablation of the protrudin gene in mice, however, gives rise to psychiatric-like disorders as a result of a loss of function, suggesting that protrudin might play an indispensable role in normal neuronal development and behavior. Protrudin forms a complex with PDZD8 as well as interacts with VAP and Rab7 at ER-endosome contacts. PDZD8 is a mammalian paralog of the ERMES subunit Mmm1, which mediates lipid transfer between the ER and mitochondria. PDZD8 in association with protrudin similarly mediates lipid transfer from the ER to endosomes and thereby contributes to endosome maturation and maintenance of neuronal integrity. The types of lipids transferred by the protrudin-PDZD8 complex *in vivo* remain to be determined. In addition, the detailed mechanism underlying lipid transfer mediated by the protrudin-PDZD8 complex at ER-endosome MCSs, including the identity of the factor or factors responsible for lipid insertion, awaits further investigation.

## Author Contributions

MS supervised the study and wrote the manuscript.

## Conflict of Interest

The author declares that the research was conducted in the absence of any commercial or financial relationships that could be construed as a potential conflict of interest.
